# Sulfur isotopes of hydrothermal vent fossils and insights into microbial sulfur cycling within a lower Paleozoic (Ordovician‐early Silurian) vent community

**DOI:** 10.1111/gbi.12495

**Published:** 2022-05-18

**Authors:** Magdalena N. Georgieva, Crispin T. S. Little, Richard J. Herrington, Adrian J. Boyce, Aubrey L. Zerkle, Valeriy V. Maslennikov, EIMF, Adrian G. Glover

**Affiliations:** ^1^ Life Sciences Department Natural History Museum London UK; ^2^ Univ. Brest, CNRS, Ifremer, UMR6197 Biologie et Ecologie des Ecosystèmes marins Profonds Plouzané France; ^3^ School of Earth and Environment University of Leeds Leeds UK; ^4^ Earth Sciences Department Natural History Museum London UK; ^5^ Scottish Universities Environmental Research Centre Glasgow UK; ^6^ School of Earth and Environmental Sciences and Centre for Exoplanet Science University of St. Andrews St. Andrews UK; ^7^ South Urals Research Center of Mineralogy and Geoecology Urals Branch of the Russian Academy of Sciences Miass Russia; ^8^ Edinburgh Ion Microprobe Facility University of Edinburgh Edinburgh UK

**Keywords:** chemosynthesis, evolution, microbiology, paleobiology, pyrite preservation

## Abstract

Symbioses between metazoans and microbes involved in sulfur cycling are integral to the ability of animals to thrive within deep‐sea hydrothermal vent environments; the development of such interactions is regarded as a key adaptation in enabling animals to successfully colonize vents. Microbes often colonize the surfaces of vent animals and, remarkably, these associations can also be observed intricately preserved by pyrite in the fossil record of vent environments, stretching back to the lower Paleozoic (Ordovician‐early Silurian). In non‐vent environments, sulfur isotopes are often employed to investigate the metabolic strategies of both modern and fossil organisms, as certain metabolic pathways of microbes, notably sulfate reduction, can produce large sulfur isotope fractionations. However, the sulfur isotopes of vent fossils, both ancient and recently mineralized, have seldom been explored, and it is not known if the pyrite‐preserved vent organisms might also preserve potential signatures of their metabolisms. Here, we use high‐resolution secondary ion mass spectrometry (SIMS) to investigate the sulfur isotopes of pyrites from recently mineralized and Ordovician‐early Silurian tubeworm fossils with associated microbial fossils. Our results demonstrate that pyrites containing microbial fossils consistently have significantly more negative δ^34^S values compared with nearby non‐fossiliferous pyrites, and thus represent the first indication that the presence of microbial sulfur‐cycling communities active at the time of pyrite formation influenced the sulfur isotope signatures of pyrite at hydrothermal vents. The observed depletions in δ^34^S are generally small in magnitude and are perhaps best explained by sulfur isotope fractionation through a combination of sulfur‐cycling processes carried out by vent microbes. These results highlight the potential for using sulfur isotopes to explore biological functional relationships within fossil vent communities, and to enhance understanding of how microbial and animal life has co‐evolved to colonize vents throughout geological time.

## 1 INTRODUCTION

Hydrothermal vents in the modern deep ocean are populated by remarkable communities of highly specialized animals, dependent on symbioses with chemosynthetic prokaryotes for their nutrition. These environments, which have existed on Earth since the Hadean (Martin et al., 2008; Russell & Hall, 1997), are also known to have been important habitats throughout geological history as evidenced by their fossil record (Campbell, 2006; Georgieva et al., 2021; Little et al., 1998). Microbes capable of oxidizing sulfide are particularly crucial to primary production within modern vent environments (Sievert et al., 2007; Sievert & Vetriani, 2012), and symbioses between metazoans and microbes involved in sulfur cycling are integral to the ability of animals to colonize and thrive at vents (Dubilier et al., 2008). For example, the tube‐dwelling annelid worm *Ridgeia piscesae* (Annelida: Siboglinidae) obtains all of its nutrition from sulfide‐oxidizing endosymbionts (Chao et al., 2007), while another vent annelid tubeworm, *Alvinella pompejana* (Annelida: Alvinellidae) associates with and grazes on diverse bacteria involved in sulfur cycling that attach to its outer body wall and tube surfaces (Cottrell & Cary, 1999; Gaudron et al., 2012; Prieur et al., 1990). Furthermore, the microbial communities associated with the dwelling tubes of modern vent annelids can also be rapidly mineralized (and hence fossilized), with a range of microbial morphotypes preserved (Buschmann & Maslennikov, 2006; Georgieva et al., 2015; Maginn et al., 2002). While it may be supposed that ancient vent animals would have also relied on microbes for their nutrition, the metabolic pathways of ancient hydrothermal vent microbes have not been explored, and it is not known if functional signals can fossilize within the sulfide minerals formed in hydrothermal vent settings.

One of the oldest hydrothermal vent communities that includes metazoans occurs within the Ordovician‐early Silurian (~440 Ma) Yaman Kasy volcanogenic massive sulfide (VMS) deposit, Ural Mountains, Russia (Little et al., 1997, 1999). Yaman Kasy VMS deposit hosts the most diverse ancient vent community known, and also demonstrates exceptional fossil preservation by pyrite including micron‐scale microbial fossils associated with the dwelling tubes of two fossil tubeworm species, *Yamankasia rifeia* and *Eoalvinellodes annulatus* (Georgieva et al., 2018). Sulfide minerals from the Yaman Kasy VMS deposit also contain elevated amounts of microbial hydrocarbons (Blumenberg et al., 2012). In non‐vent settings in which fine‐grained pyrite formation occurs, stable isotopes of sulfur are frequently employed to investigate the metabolic strategies of both modern and fossil organisms, such as to detect the sulfate‐reducing metabolisms of Precambrian fossil microbial filaments (Schopf et al., 2015; Wacey et al., 2011). Microbial sulfate reduction and microbial sulfur disproportionation can result in large δ^34^S fractionations of 30‰–40‰ (Canfield, 2001; Detmers et al., 2001), while the effects of sulfide oxidation on sulfur isotope fractionation are smaller (Glynn et al., 2006). Phototrophic oxidation of hydrogen sulfide to elemental sulfur typically results in a small inverse isotope effect (of +1.8 ± 0.5‰), while oxidation of elemental sulfur to sulfate produces a small normal isotope effect (of −1.9 ± 0.8‰) (Zerkle et al., 2009). Sulfur isotope fractionations that result from chemotrophic sulfide oxidation (up to +8‰) are also distinct from those produced during abiotic oxidation of sulfide with oxygen (∼−5‰) (Zerkle et al., 2016), and can in some cases produce much larger effects (of up to +12.5‰) (Pellerin et al., 2019), suggesting it may therefore also be possible to detect signatures of sulfide oxidation in the fossil record.

Sulfur isotopes of pyrite have also been explored within both modern and ancient hydrothermal vent environments from a range of tectonic settings. δ^34^S values of 0 to +6‰ are typical for sulfide minerals from non‐sedimented, Phanerozoic hydrothermal vents (Franklin et al., 2005; Huston, 1999; Seal, 2006). δ^34^S values for sulfides in modern systems generally reflect that of vent fluid hydrogen sulfide (H_2_S) from which the minerals precipitated; sulfide minerals that are in equilibrium with vent fluid H_2_S typically have δ^34^S values within −2‰ to +3‰ of vent fluid H_2_S, at temperatures of 100–400°C (Ohmoto & Rye, 1979; Shanks et al., 1995). At equilibrium conditions, pyrite should have δ^34^S values greater than that of associated vent fluid, but in practice, the δ^34^S values are often lighter, possibly as a result of precipitation from lower temperature fluids (Rouxel et al., 2004) or via thiosulfate intermediates (Ono et al., 2007). δ^34^S values of hydrothermal vent sulfide minerals below approximately −4‰ have been interpreted to indicate contributions from microbial sulfate reduction (MSR) (Ding et al., 2021; Eickmann et al., 2020; Peters et al., 2010). MSR is considered to be a particularly important process at sedimented hydrothermal vents (Fowler et al., 2019; McDermott et al., 2015) and in the hydrothermal vent subsurface, where it occurs during the low‐temperature alteration of oceanic crustal rocks (Ono et al., 2012; Rouxel et al., 2004). Apart from several δ^34^S values collected through isotope ratio mass spectrometry for tubeworm fossils from the Yaman Kasy VMS deposit (−2.5‰ to +0.5‰; Herrington et al., 1998) and the Carboniferous Ballynoe deposit, Silvermines, Ireland (−23.2‰ to −18.4‰; Boyce et al., 2003), the sulfur isotope values of hydrothermal vent fossils, both ancient types and those forming in the modern ocean, have been largely unexplored. In addition, sulfur isotopes of seafloor hydrothermal vent sulfide minerals are rarely explored in high spatial resolution (at scales of ≤5 μm), but at this scale, sulfur isotope measurements have the power to resolve sulfur cycling processes related to distinct mineral texture types.

Here, we use high‐resolution secondary ion mass spectrometry (SIMS) to explore for the first time the fine‐scale δ^34^S signatures of pyrite delineating fossilized vent animals and associated microbes from both modern and Phanerozoic sediment‐free hydrothermal vent environments. We discuss our observations in the context of pyrite preservation and the nature of sulfur‐based metabolic pathways that contributed to pyrite formation. We suggest that δ^34^S values of pyrite containing microbial fossils are indicative of sulfur‐cycling metabolisms of microbes from both recent and Ordovician‐early Silurian hydrothermal vent communities.

## 2 GEOLOGICAL BACKGROUND

Recent samples used in this study comprised two mineralized tubes of two hydrothermal vent tubeworm species, *Alvinella* sp. from the 9°50′N segment of the East Pacific Rise (Bio9 vent), and *Ridgeia piscesae* tubes from the Endeavour vent site, Juan de Fuca Ridge, northeast Pacific (Figures S1 and S2 in File S1). The East Pacific Rise is a fast‐spreading mid‐ocean ridge, with the 9°50′N segment being highly active, several volcanic eruptions have been documented here to date (Fornari et al., 2012). Both high‐temperature vents (with fluids of over 50 to ~410°C) and moderate‐ to low‐temperature vents (with fluids <30°C) occur on the 9°50’N segment (Hessler et al., 1985), while hydrogen sulfide concentrations of high‐temperature vents are typically up to 1500 μM, and 100–300 μM at diffuse vents (Le Bris et al., 2006; Le Bris & Gaill, 2007). *Alvinella* spp. worms typically colonize the surfaces of high‐temperature vents, where the dwelling tubes of these annelids are exposed to high rates of mineralization (Georgieva et al., 2015). The Endeavour segment of the Juan de Fuca Ridge is an intermediate‐spreading mid‐ocean ridge, with less‐frequent eruptions and a magma‐driven system that distinctly varies from that which occurs beneath the East Pacific Rise (Kelley et al., 2012). Large hydrothermal chimneys of over 30 m height are common at this site, and in general, hydrothermal fluids are enriched in methane and ammonia, unusual for a mid‐ocean ridge vent. Vent fluid temperatures at the Main Endeavour Field can be up to 402°C, while proximal diffuse‐flow sites are common. *R. piscesae* occurs at high densities at Endeavour and can colonize a range of vent conditions and exhibit a diversity of morphotypes (Tunnicliffe & Kim Juniper, 1990). The mineralization of the tubes of annelids has also been documented at this site (Cook & Stakes, 1995).

Ancient fossil samples used in this study consisted of two tubes from the late Ordovician‐early Silurian Yaman Kasy VMS deposit (Figures S1, S3 in File S1), described as the fossil species *Yamankasia rifeia* and *Eoalvinellodes annulatus* (Little et al., 1999). The Yaman Kasy VMS deposit occurs in the southern Ural Mountains, Russia and is considered to be early Silurian to late Ordovician in age (Buschmann & Maslennikov, 2006). This deposit exhibits intricate preservation of hydrothermal vents that likely formed within a back‐arc basin (Herrington, Puchkov, et al., 2005). Vent chimneys and the most diverse ancient vent community are preserved as part of this deposit (Georgieva et al., 2021; Herrington et al., 1998), which has been extensively discussed in scientific literature due to the important insights it provides into ancient hydrothermal vent environments (Ayupova et al., 2017; Buschmann & Maslennikov, 2006; Georgieva et al., 2021; Herrington et al., 1998; Herrington, Maslennikov, et al., 2005; Kuznetsov et al., 1993; Little et al., 1997, 1999; Maslennikov et al., 2017).

## 3 METHODS

Details of the four fossil tube samples selected for this study are listed in Table 1. Transverse and longitudinal sections of tube walls were prepared into polished blocks. Polished block sample images were collected using both reflected light (RL) and scanning electron microscopy (SEM) in backscatter electron mode to identify mineral phases and select targets for sulfur isotope measurements. RL microscopy was performed using a Zeiss AxioImager M2 microscope, and SEM using a FEI Quanta 650 FEG‐SEM, both at the Natural History Museum, UK. Polished blocks were coated with carbon (approximately 10 nm thickness) prior to SEM. Pyrite was the only mineral phase selected for isotopic analysis, as it is the main mineral phase found to be preserving hydrothermal vent fossils (aside from silica and occasionally marcasite, zinc sulfides (sphalerite and/or wurtzite), and minor quantities of copper containing sulfides (chalcopyrite and isocubanite) observed in Georgieva et al., 2015, 2018). Pyrite is also the main mineral phase found to preserve especially fine structures such as microbes (Georgieva et al., 2015, 2018; Little et al., 1997). Pyrite texture types targeted for analysis were based on the main textures observed within the specimens: pyrite containing microbial fossils, and pyrite without microbial fossils and exhibiting either a predominantly colloform, porous, or smooth texture. Microbial fossils in hydrothermal vent pyrites are usually apparent as hollow filaments approximately 1 μm in diameter, present in a range of orientations, and exhibiting curved morphologies and occasionally “cell” shapes typical of bacteria such as septate divisions within filaments and rod‐like morphologies (Georgieva et al., 2015, 2018). High‐resolution images of pyrite containing microbial fossils in our samples are presented in Figures S2 and S3 in File S1.

**TABLE 1:** Information on samples used during the current study. Minimum, maximum, and mean δ^34^S values (‰) are given for pyrite measurements illustrated in Figure 3

Sample	Code	Age	Description	Deposit	Pyrite texture of tube wall	Minimum δ^34^S (‰)	Maximum δ^34^S (‰)	Mean δ^34^S (‰)	2SD δ^34^S (‰)	*n*
P23671	M72, Alvin 4375 CG3‐4 Bio9	Recent	*Alvinella* sp. annelid tube	East Pacific Rise, Eastern Pacific	With microbial fossils	−7.1	−0.1	−3.6	4.3	14
					Colloform, no microbial fossils	−1.5	0.7	−0.5	1.7	8
P23672	Ropos 278 94ENDV‐11	Recent	*Ridgeia piscesae* annelid tube	Juan de Fuca Ridge, North‐East Pacific	With microbial fossils	−6.8	−1.2	−4.2	3.0	13
					Porous, no microbial fossils	−3.7	−0.2	−1.6	2.3	10
					Smooth, no microbial fossils	0.5	2.0	1.4	0.9	8
P23934	NHMUK OR6468	Ordovician‐early Silurian	*Yamankasia rifeia* worm tube	Yaman Kasy, Southern Ural Mountains	With microbial fossils	−9.3	−1.5	−4.1	4.3	28
					Smooth, no microbial fossils	−4.4	0.4	−1.4	2.6	18
P23935	YKB	Ordovician‐early Silurian	*Eoalvinellodes annulatus* worm tube	Yaman Kasy, Southern Ural Mountains	With microbial fossils	−6.9	−1.4	−4.7	3.0	28
					Porous, no microbial fossils	−4.8	0.9	−2.0	3.8	15

For sulfur isotope analyses, samples were re‐mounted into polished blocks approximately 25 mm in diameter and 8 mm thick, with a small grain of Balmat pyrite standard (from the Balmat Mine, New York, USA; crystal supplied by J. Valley, University of Wisconsin‐Madison) (Crowe et al., 1990) embedded close to the targets selected for isotopic analysis within each specimen. Blocks were cleaned in an ultrasonic bath with deionized water (for 1 min) then ethanol (for 30 s), dried thoroughly, and coated with gold (approximately 30 nm thickness). The samples were then introduced into the specimen exchange chamber of a Cameca 1270 secondary ion mass spectrometry (SIMS) instrument at the Ion Microprobe Facility, University of Edinburgh, UK, leaving them for at least 18 h to achieve the vacuum conditions necessary for analysis. For analytical measurements, a Cs ion source microbeam with a current of 1 nA was focused into an ellipse a maximum of approximately 7 μm wide on the surface of the sample.

Samples were pre‐sputtered for 30 s using a raster of size of 20 μm to remove the gold coat, the secondary ion beam was then automatically aligned into the center of the field aperture, and subsequently, 20 cycles of 4 s were used to collect secondary ions. ^32^S and ^34^S were collected simultaneously using Faraday cup detectors. To monitor analytical precision, sample analyses were interspersed with measurements of Balmat pyrite standard, collecting approximately 5 standard measurements per 10–15 sample measurements. Balmat pyrite measurements were also used to correct for instrument bias, which was computed for each sample by calculating the slope (m) and intercept (c) of Balmat pyrite ^34^S/^32^S results, and adjusting raw δ^34^S values according to the Balmat calibration line. For example in Table S1, Std@t = (^34^S/^32^S)m + c, and this is converted to a corrected δ^34^S value using the formula δ^34^S corr. = (((^34^S/^32^S)/Std@t)*(14.63 + 1000) − 1000). δ^34^S results are reported relative to the Vienna Canyon Diablo Troilite scale (V‐CDT; Ding, Valkiers, et al., 2001). Further details of the analytical conditions of the Cameca 1270 SIMS used for analysis are provided in File S2.

After SIMS analyses, the samples were coated with a thin layer of gold (approximately 5 nm thickness) and again imaged using SEM to assess the sizes of analytical pits (and hence verify the resolution of measurements), and to verify their locations (which pyrite texture type had been measured). Based on these observations, the pyrite textural category of each SIMS measurement was checked. Sulfur isotope data (File S3) were subsequently analyzed in R (R Core Team, 2013) using the *tidyverse* package bundle (Wickham et al., 2019), with full results of each statistical test employed outlined in File S4. Shapiro–Wilk tests were used to test for normality of δ^34^S measurements of target pyrite texture types within each sample, while a *F*‐tests were used to determine whether the variances of δ^34^S measurements differed between pairs of target pyrite texture types within each sample. *T*‐tests were employed to assess whether the means of δ^34^S values significantly differed between pairs of target pyrite texture types within each sample. As one of the datasets failed the assumption of normality required for the *t*‐test, and several datasets were found to have unequal variances, the nonparametric Wilcoxon test was also performed to determine whether distributions of δ^34^S values significantly differed between pairs of target pyrite texture types within each sample.

## 4 RESULTS

### 4.1 Sample characterization

**FIGURE 1:**
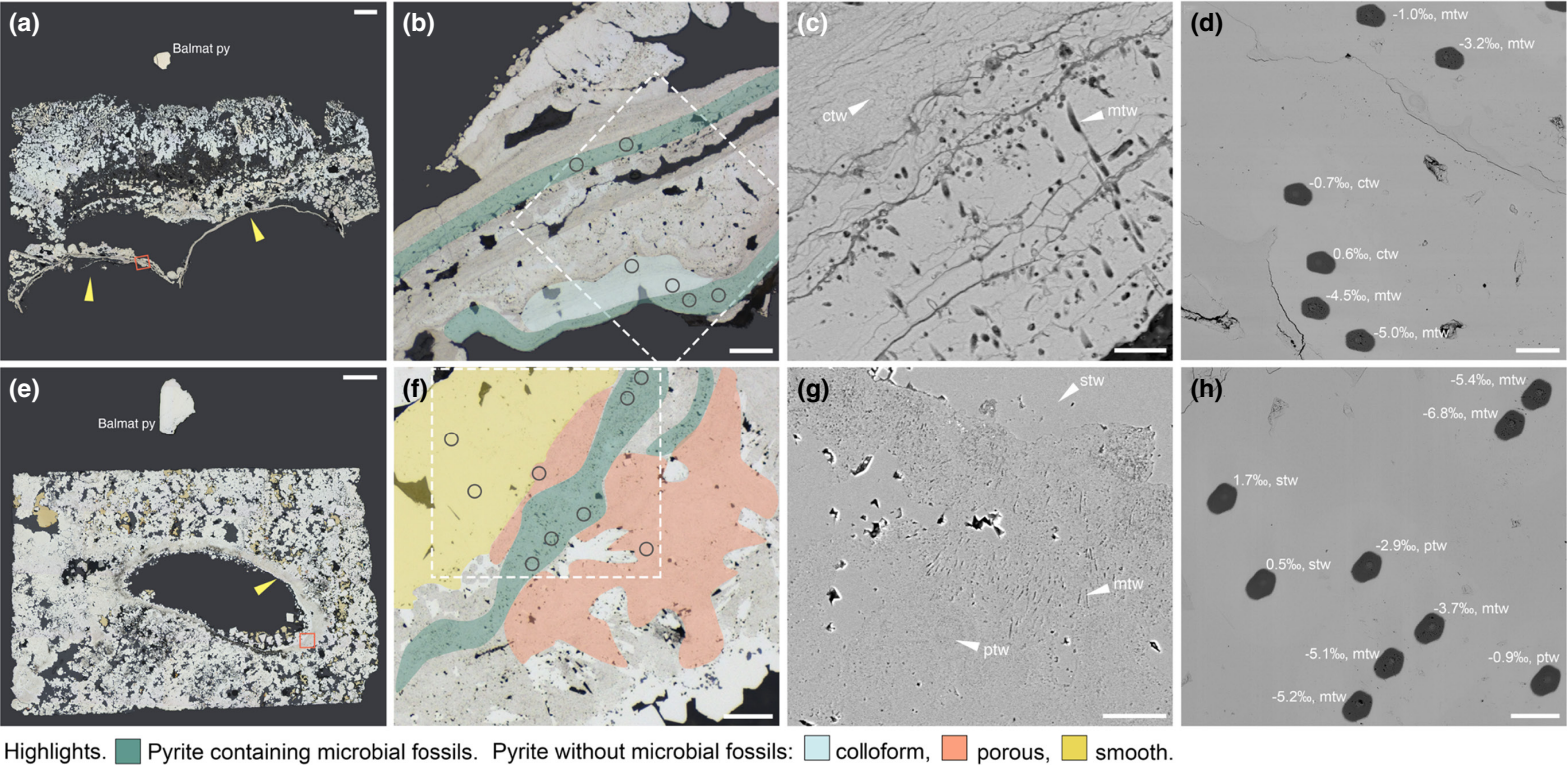
Recently mineralized annelid tubes from modern vent environments. (a–d) *Alvinella* sp. specimen P23671. (a) RL image of partial transverse sections of two tubes, yellow arrows point to tube walls. Orange box shows location of B, scale bar is 1 mm. (b) RL image of the mineralized tube wall, showing preservation by colloform pyrite and the presence of filamentous microbes with these zones highlighted, scale bar is 50 μm. Dashed box shows location of (d), and circles delineate locations of sulfur isotope measurements. (c) SEM image showing detail of microbial fossils and adjacent pyrite of a colloform texture without microbial fossils, scale bar is 5 μm. (d) SEM image of SIMS pits following sulfur isotope analysis with δ^34^S results (‰) and pyrite texture type annotated, scale bar is 30 μm. (e–h) *Ridgeia piscesae* specimen P23672. (e) RL image of transverse section of a tube, yellow arrow points to tube wall. Orange box shows location of F, scale bar is 1 mm. (f) RL image of mineralized tube wall showing preservation by pyrite exhibiting porous and smooth textures and the presence of filamentous microbes with these zones highlighted, scale bar is 50 μm. Dashed box shows location of (h), and circles delineate locations of sulfur isotope measurements. (g) SEM image showing detail of microbial fossils and pyrite exhibiting porous and smooth textures, scale bar is 20 μm. (h), SEM image of SIMS pits following sulfur isotope analysis with δ^34^S results (‰) and pyrite texture type annotated, scale bar is 30 μm. ctw, colloform tube wall; mtw, microbial tube wall; ptw, porous tube wall; stw, smooth tube wall

**FIGURE 2:**
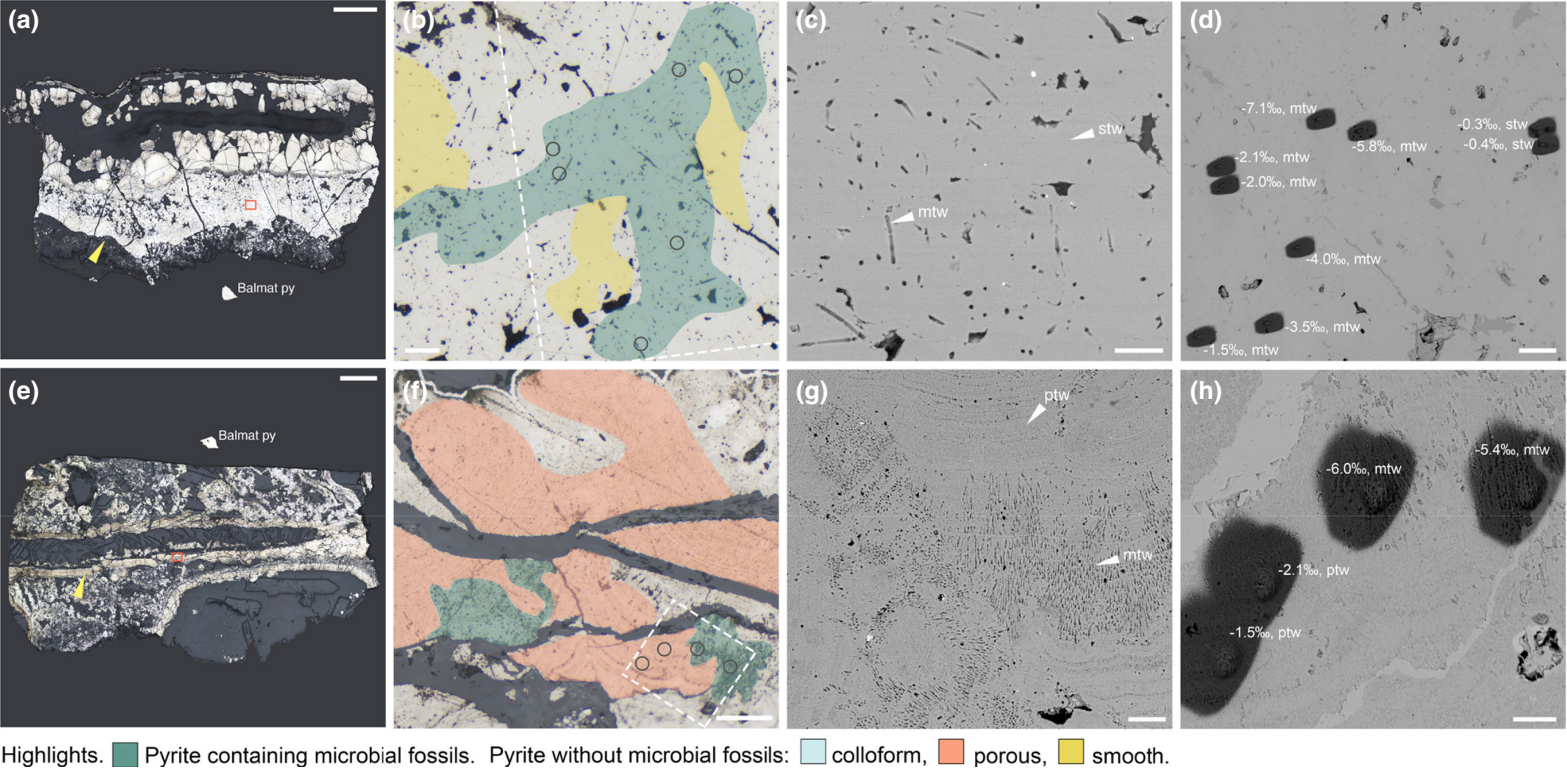
Ordovician‐early Silurian fossil worm tubes from the ancient hydrothermal vent deposit Yaman Kasy. (a–d) *Yamankasia rifeia* specimen P23934. (a) RL image of a longitudinal tube section with a thick wall, indicated by yellow arrow. Orange box shows location of (b), scale bar is 2 mm. (b) RL image of the mineralized tube wall, showing preservation by smooth pyrite and the presence of numerous microbes, with these zones highlighted, scale bar is 20 μm. Dashed box shows partial location of (d), and circles delineate locations of sulfur isotope measurements. (c) SEM image showing detail of microbial fossils and adjacent smooth pyrite without microbial fossils, scale bar is 10 μm. (d) SEM image of SIMS pits following sulfur isotope analysis with δ^34^S results (‰) and pyrite texture type annotated, scale bar is 30 μm. (e–h) *Eoalvinellodes annulatus* specimen P23935. (e) RL image of a longitudinal tube section, yellow arrow points to tube wall and orange box shows location of F, scale bar is 2 mm. (f) RL image of the mineralized tube wall showing preservation by pyrite exhibiting a porous texture and the presence of filamentous microbes with these zones highlighted, scale bar is 50 μm. Dashed box shows location of H, and circles delineate locations of sulfur isotope measurements. (g), SEM image showing detail of microbial fossils and pyrite exhibiting a predominantly porous texture, scale bar is 20 μm. (h) SEM image of SIMS pits following sulfur isotope analysis with δ^34^S results (‰) and pyrite texture type annotated, scale bar is 10 μm. ctw, colloform tube wall; mtw, microbial tube wall; ptw, porous tube wall; stw, smooth tube wall

RL microscopy revealed that in all of our samples (Table 1), fossil tube walls are delineated by pyrite (Figures 1a,b,e,f and 2a,b,e,f). A number of these fossils, notably recently fossilized *Alvinella* sp. and *R. piscesae* annelid tubes and Ordovician‐early Silurian tube fossils from the Yaman Kasy VMS deposit, contain microbial fossils which are apparent as filament “ghosts” delineated by pyrite (Figures 1b,c,f,g and 2b,c,f,g) as documented in Georgieva et al. (2015, 2018). Similar to other findings of fossil microbes associated with tube fossils at hydrothermal vents, the microbes are preserved as hollow filament molds approximately 1 μm diameter and delineated by fine‐grained pyrite, but largely lacking intra‐filament details (Figures 1c,g and 2c,g).

The texture of pyrite preserving vent macrofossils and microbes generally varies by sample. *Alvinella* sp. tube walls are preserved mainly by colloform pyrites with a smooth, even surface in section that do not exhibit a high porosity. Different generations of pyrite could be discerned by the layering of pyrite horizons with slight variations in color (Figure 1b). Microbe fossils occur within some of these layers, with individual microbial fossils at times spanning across two layers (Figure 1c). *Ridgeia piscesae* tubes and associated microbes are preserved by pyrites with a predominantly porous appearance, which is generally flanked by later overgrowth of smooth pyrites that also occur as localized inclusions within regions of porous pyrite (Figure 1f,g). Porous pyrites also contain remnant colloform/botryoidal textures (Figure 1g), but layering is far less discernable in comparison with *Alvinella* sp. tube samples. The Ordovician‐early Silurian *Yamankasia rifeia* tube fossil and microbe sample was preserved by smooth pyrites (Figure 2b,c) that occurred as a thick layer which comprised the fossil tube wall (Georgieva et al., 2018). Microbial fossils formed diffuse clusters within this pyrite, with no clear demarcation between regions of pyrite that contained microbial fossils and those that did not. On the other hand, the Ordovician‐early Silurian *Eoalvinellodes annulatus* tube fossil and microbe sample was preserved by pyrite of a predominantly porous texture (Figure 2f,g) in which colloform/botryoidal growth is apparent. Microbial filament fossils occurred as distinct, localized clusters within pyrite of this texture, which as in the *Alvinella* sp. sample were observed to crosscut layering.

### 4.2 Sulfur isotopes of vent fossils

**FIGURE 3:**
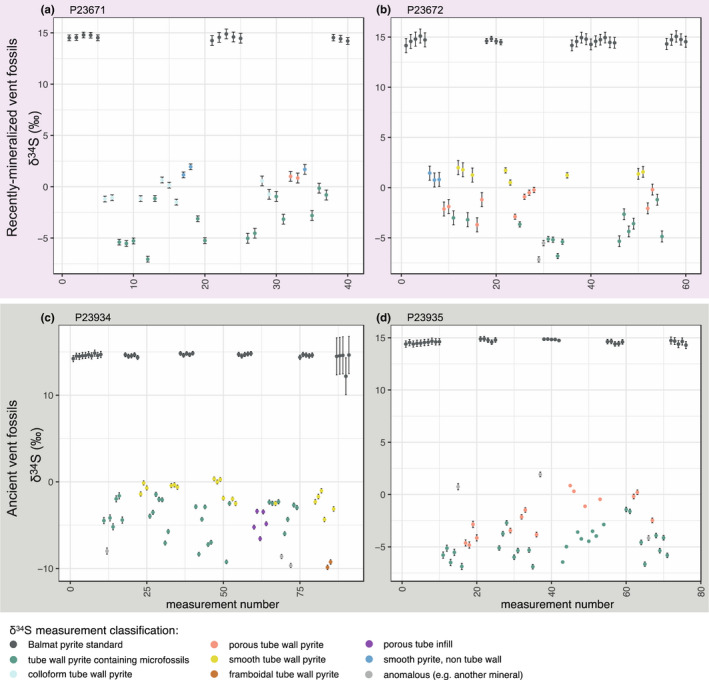
Results of all SIMS δ^34^S measurements within each analyzed specimen. (a,b) recently mineralized annelid tubes from modern vent environments, (c,d) Ordovician‐early Silurian fossil worm tubes from the ancient hydrothermal vent deposit Yaman Kasy. Points are colored by the mineral and/or textural classification of each measurement, and error bars represent 2SD. (a) recently mineralized *Alvinella* sp. tubes. (b) recently mineralized *Ridgeia piscesae* tubes. (c) Ordovician‐early Silurian *Yamankasia rifeia* tube. (d) Ordovician‐early Silurian *Eoalvinellodes annulatus* tube

**FIGURE 4:**
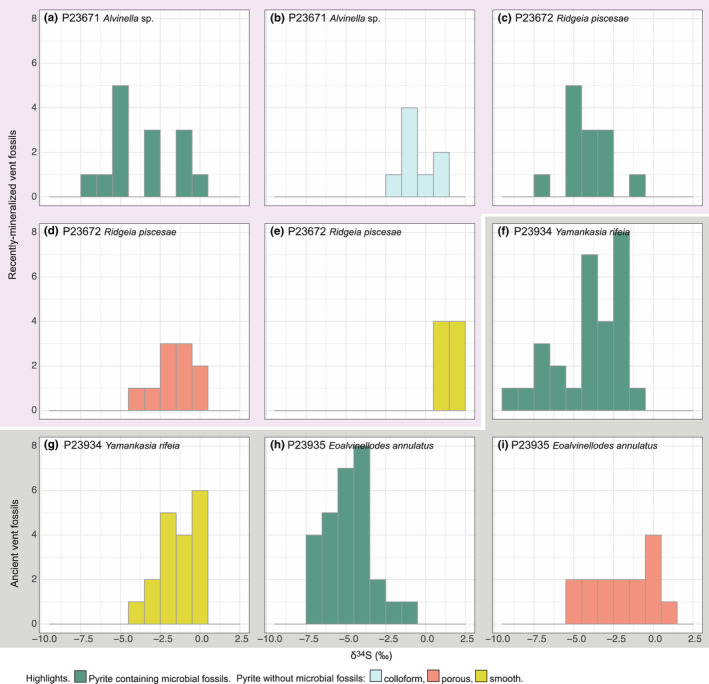
Histograms of SIMS δ^34^S measurements for each target pyrite texture type within each sample. (a,b) recently mineralized *Alvinella* sp. tubes. (a–e) recently mineralized *Ridgeia piscesae* tubes. (f,g) Ordovician‐early Silurian *Yamankasia rifeia* tube from the Yaman Kasy VMS deposit. (h,i) Ordovician‐early Silurian *Eoalvinellodes annulatus* tube from the Yaman Kasy VMS deposit

**FIGURE 5:**
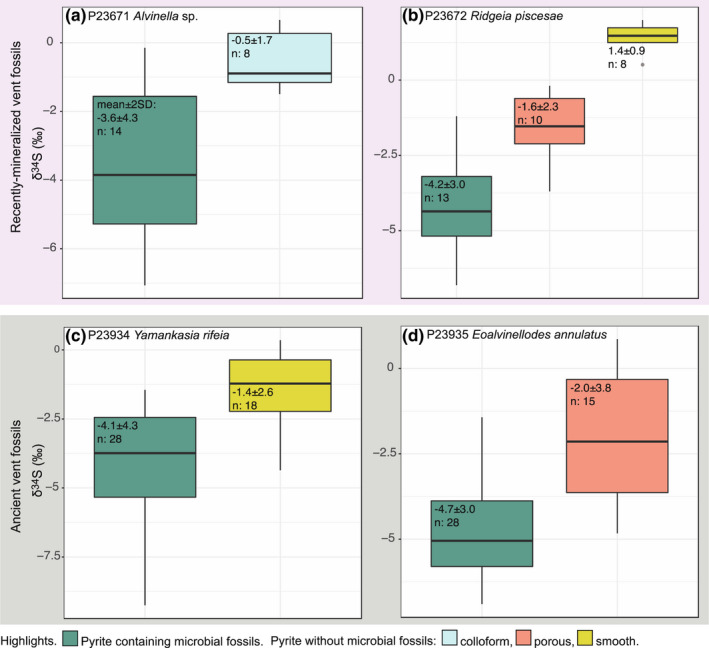
Boxplots demonstrating SIMS δ^34^S measurements of target pyrites within each analyzed specimen; (a,b) recently mineralized annelid tubes from modern vent environments, (c,d) Ordovician‐early Silurian fossil worm tubes from the ancient hydrothermal vent deposit Yaman Kasy. Boxplots are colored by pyrite texture type, with the mean, two standard deviations, and number of measurements indicated on boxplots. (a) recently mineralized *Alvinella* sp. tubes. (b) recently mineralized *Ridgeia piscesae* tubes. (c) Ordovician‐early Silurian *Yamankasia rifeia* tube. (d) Ordovician‐early Silurian *Eoalvinellodes annulatus* tube

A total of 156 sulfur isotope analyses of target pyrite textures in hydrothermal vent samples, and 102 measurements of Balmat pyrite standard were performed on 4 samples (Figure 3; File S3). The standard error (1×) of the mean of Balmat pyrite δ^34^S corrected measurements in each sample varied between 0.01‰ and 0.16‰ (this value includes counting errors and other discrepancies such as sample movement, unevenness of the sample surface, and operation of the electron gun). δ^34^S analyses were focused mainly on pyrite containing microbial fossils, and pyrite of the same or similar generation directly adjacent to pyrite containing microbial fossils that did not contain microbial fossils (Figures 4 and 5). However, due to the limited resolution of the camera within the SIMS instrument, this was not always possible and meant it is essential to check the location of the SIMS pit using SEM following sulfur isotope analyses. SEM imaging of SIMS pits (Figures 1d,h and 2d,h) further revealed that the resolution achieved for δ^34^S analyses was approximately 5–7 μm. As the presence of pyrite texture types was found to vary between samples, and due to observations of sulfur isotope variation on very small scales (see later), δ^34^S data for each sample were analyzed individually and thus fossil and non‐fossil pyrite isotope values were only compared within the same sample. The data were filtered to remove measurements of non‐target mineral phases and pyrite textures.

Sulfur isotopic compositions of the analyzed pyrite grains representing recently mineralized annelid tubes and associated microbes from modern vent environments (Figure 1) show statistically distinct differences in δ^34^S between tube wall pyrite containing microbial fossils, and colloform tube wall pyrite in which microbial fossils were absent for *Alvinella* sp. (*t*‐test *p*‐value: <0.001; Wilcoxon test *p*‐value: <0.005) (Figures 4a,b and 5a). δ^34^S values of colloform pyrite are close to 0‰ (±1.7‰ 2SD, *n* = 8), while those of pyrite with microbial fossils are significantly more depleted in ^34^S by on average 3‰ (mean of −3.6‰, ±4.3‰ 2SD, *n* = 14) (Figure 5a). In some instances, δ^34^S measurements of the different pyrite textures taken ~20 μm from each other demonstrate differences of ~4‰ (Figure 1d). Pyrites containing microbial fossils are also more depleted in ^34^S within the mineralized tubes of *Ridgeia piscesae*, and exhibit a similar range to that observed within *Alvinella* sp. tubes (Figures 4c–e and 5b). The δ^34^S values of microbial pyrite observed for *R. piscesae* tubes overlap with those recorded for nearby porous pyrite lacking microfossils. However, a *t*‐test confirmed a significant difference between the two different pyrite groups (*t*‐test *p*‐value: <0.001; Wilcoxon test *p*‐value: <0.001). δ^34^S values of smooth pyrites within *R. piscesae* samples do not overlap with microbially textured pyrites, being also significantly different (*t*‐test *p*‐value: <0.001; Wilcoxon test *p*‐value: <0.001), and centered on values of approximately +1.4‰ (±0.9‰ 2SD, *n* = 8). Thus, in all cases of modern organisms, there is a statistically distinct ^34^S depletion in microbially populated pyrite, compared with non‐fossil‐bearing textures.

Results for Ordovician‐early Silurian tube fossils from the Yaman Kasy VMS deposit containing microbial fossils reflect those for mineralized tubes from modern vent environments. Again, in the *Yamankasia rifeia* tube sample (Figures 4f,g and 5c), microbially textured pyrites report a statistically robust ^34^S‐depletion compared with adjacent smooth pyrites (Wilcoxon test *p*‐value: <0.001). Microbial pyrites in this ancient sample exhibit a large range in δ^34^S, from −9.3‰ to −1.5‰, and overlap with the lowest values for smooth pyrite. Results for the *Eoalvinellodes annulatus* tube sample also demonstrate the same pattern of lower δ^34^S values for microbial pyrite compared with pyrite that does not contain microbial fossils (Figures 4h–i and 5d), and while ranges for the two pyrite types analyzed within this sample overlap, the difference in δ^34^S between the two is again statistically significant (*t*‐test *p*‐value: <0.001; Wilcoxon test *p*‐value: <0001), in both cases mimicking the modern examples.

Our results also demonstrate differences between pyrite types that do not contain microbial fossils in the vent fossil samples analyzed. In mineralized *Ridgeia piscesae* tubes (Figure 1e–g), pyrite of a porous texture and smooth pyrite have significantly different δ^34^S values (*t*‐test *p*‐value: <0.001; Wilcoxon test *p*‐value: <0.001), with porous pyrite being lower in δ^34^S by approximately 2‰–3‰.

## 5 DISCUSSION

Recent research has shown that that hydrothermal vents are important preservational settings where mineral precipitation can readily fossilize resistant biological structures (from those made by macrofauna to microbes), at times with very fine detail and at sub‐micron scales (Georgieva et al., 2015, 2018, 2021; Maginn et al., 2002; Maslennikov et al., 2017). In addition, hydrocarbons of microbial origin may be preserved alongside vent sulfides for hundreds of millions of years (Blumenberg et al., 2012). As such, these environments provide a crucial glimpse into ancient deep‐sea communities driven by chemosynthesis. In our study, we are confident that the pyrite we analyzed formed rapidly, preserving the hydrothermal vent fossils without later recrystallization or mineral replacement. As a result, the pyrite δ^34^S values that we have recorded here have not been affected by diagenesis and thus are likely to reflect primary formation conditions (Georgieva et al., 2015, 2021). While there are emerging new studies on pyrite formation and precipitation pathways and the various controls on its sulfur isotope composition at vents (e.g., Findlay et al., 2019), studies of how these processes interact with biological structures are rare. It has been suggested that organisms can promote the precipitation of sulfide minerals at vents (Maginn et al., 2002; Peng et al., 2007; Zbinden et al., 2001) and affect pyrite texture (Georgieva et al., 2015), but apart from observations of increased phosphorus content in a mineralized annelid tube wall (Maginn et al., 2002) as of yet there are no indications that pyrite chemistry is also affected. This study, in which sulfur isotopes of vent samples are explored at scales of ~5 μm, firstly demonstrates significant variation of sulfur isotopes within hydrothermal vent pyritized fossils at distances of as little as 20 μm. Variations in δ^34^S of up to 9‰ are observed and are directly associated with the presence of microbial fossils (for example, see Figure 2h). In addition, whilst a limited overlap of datasets is seen, there is clearly a significant difference in δ^34^S between pyrite containing microbial fossils, which are always isotopically depleted in ^34^S compared to nearby non‐fossiliferous pyrite for all samples, whether ancient or modern (Figure 5). Clearly, the presence or absence of microbial fossils influences the sulfur isotope signature of pyrites with different textures.

The range of pyrite sulfur isotope values reported here for mineralized *Alvinella* sp. and *Ridgeia piscesae* tubes are also quite different than those reported from most other modern vent sulfide samples, such as those described by Ono et al. (2007; +0.4‰ to +3.4‰) from the East Pacific Rise and Rouxel et al. (2004; −0.5‰ to +3.9‰) from Lucky Strike on the Mid‐Atlantic Ridge. In the latter study, δ^34^S values of −0.5‰ were deemed to be among the lowest reported for seafloor hydrothermal deposits in non‐sedimented mid oceanic ridges, and to possibly result from microbial sulfur cycling (Rouxel et al., 2004). In contrast, our δ^34^S results are as low as −7‰ (Table 1). They are within the range of values reported by Ding et al. (2021) from the Southwest Indian Ridge, where pyrite δ^34^S values of −3.6‰ to −23.8‰ were interpreted to suggest microbial sulfate reduction. Thus, our results also indicate a potential biological influence on pyrite sulfur isotope composition. For ancient tube fossils, our pyrite δ^34^S results for the Yaman Kasy VMS deposit tubes are in agreement with the results of Herrington et al. (1998), but have higher δ^34^S values than those obtained by Boyce et al. (2003), of fossil tubes and their coatings from the Carboniferous Ballynoe deposit (δ^34^S of −23.2‰ to −18.4‰). However, the geological setting of the latter is deemed to be different from these mid‐ocean ridge VMS settings, possibly being within an intra‐cratonic extensional basin without volcanic association. Given the starkly different δ^34^S values obtained from Ballynoe pyrite in that can be as low as −40‰, they are likely not directly comparable with our data.

There are several possible abiotic mechanisms through which the intriguing sulfur isotope signatures recorded in this study could have arisen. It is conceivable that the differences between δ^34^S of microbial‐textured pyrite and pyrite without microfossils could be due to different pyrite generations with different sulfur isotope characteristics having been measured (for example, resulting from changes in the δ^34^S value of vent fluid). While this is plausible for δ^34^S differences between smooth and porous pyrite in the *Ridgeia piscesae* sample (Figures 1 and 5b) in which the smooth pyrite presents a later stage overgrowth, this is unlikely for the majority of measurements as the pyrite generation either appears to be the same (as in Figure 1b,d), or were taken from very small areas of sample. Therefore, given the rapid mineralization that would have been needed for preservation (e.g., Georgieva et al., 2015), it is more likely that the different pyrite textures measured resulted from the interaction of organisms (their structures and/or metabolisms) with vent fluids and precipitating minerals. Previous studies reporting sulfur isotope results of abiogenic vent pyrite from the East Pacific Rise (Ono et al., 2007; +0.4‰ to +3.4‰) and Lucky Strike on the Mid‐Atlantic Ridge (Rouxel et al., 2004; −0.5‰ to +3.9‰) would have likely captured a range of pyrite generations, but do not report sulfur isotope values as low as those recorded for pyrite containing microbial fossils in this study. While the above studies also suggest that disequilibrium pyrite precipitation conditions at vents can result in more ^34^S‐depleted signatures, the δ^34^S values reported here for pyrite containing microbial fossils are much lower than in the above reports, suggesting an influence beyond abiogenic pyrite precipitation dynamics. In addition, the sulfur isotope variations that we observed between different pyrite textures are unlikely to be due to a different mineral phase having been measured, as the mineralogy of all samples was carefully assessed using RL microscopy (to check for marcasite) and SEM. There are also as yet no accounts of marcasite finely preserving vent fossils, while microbial, colloform, and porous mineral textures are widely associated with pyrite growth in vent samples (Georgieva et al., 2015; Maginn et al., 2002). Any SIMS analysis spots that appeared to be on a phase other than pyrite were filtered from analyses, on the basis of SEM observations of SIMS spot locations. Thermochemical sulfate reduction, during which sulfate is reduced to sulfide (Machel, 2001), may also result in ^34^S‐depleted sulfur phases without microbial involvement, resulting in kinetic isotopic fractionations up to 17‰ (Meshoulam et al., 2016). Studies of thermochemical sulfate reduction in modern hydrothermal systems have shown that hydrothermal pyrites formed from entrained seawater sulfate are actually slightly heavier in ^34^S (e.g. Petersen et al., 2020). Given the association between negative δ^34^S values and microbial fossils in our samples, we suggest that the above process is a less likely mechanism to explain our observations.

A more reasonable explanation for the data is that the ^34^S‐depleted sulfur isotope signatures of pyrite containing microbial fossils in our samples are due to microbial fractionation effects, given the microbial fossils that it has entombed. The occurrence of sulfide minerals even in areas without observable microbial fossils strongly suggests that hydrothermal H_2_S provided the dominant source of sulfur for pyrite formation processes. The δ^34^S of the original vent fluid supplying hydrogen sulfide to the study sites is unknown, but it would be reasonable to assume a value of 0‰ to +6‰ in line with values reported for a range of modern vent sites (Seal, 2006). For 9–10°N on the East Pacific Rise from which our *Alvinella* sp. sample was collected, δ^34^S values of +4.4‰ to +5.8‰ for hydrogen sulfide were reported by Ono et al. (2007). These values are also compatible with a range of ancient vent sites from throughout the Phanerozoic (Seal, 2006). Alternatively, the δ^34^S values of non‐tube wall smooth pyrite (ranging from +0.8‰ to +2.0‰ in the *Alvinella* sp. and *Ridgeia piscesae* samples; Table S1) could be taken to represent end‐member vent fluids that have not been biologically influenced at the time of deposition. In either case, pyrite containing microbial fossils (which ranges in δ^34^S from −9.3‰ to −0.1‰) in all of our samples (Figure 5; Table 1) represents a significant ^34^S depletion from the δ^34^S of vent fluid hydrogen sulfide, assuming this would have been the dominant source of sulfur to the system.

These fractionations in δ^34^S are consistent with the upper limit of ^34^S isotope fractionations produced by MSR (of ~ −4‰). In particular, hydrogen could have been an important component of vent fluids as even basalt‐hosted vents that typically have low hydrogen vent fluid concentrations (Ding, Seyfried, et al., 2001) host microbes that can metabolize hydrogen (Adam & Perner, 2018). Hydrogenotrophic sulfate reducers generally produce very small sulfur isotope fractionations between sulfate and sulfide (of +1‰ to +6‰; Hoek et al., 2006), and thus of a similar magnitude to our results. Alternatively, heterotrophic MSR by (hyper)thermophiles at high/optimal temperatures and correspondingly high cell‐specific sulfate reduction rates also results in very small fractionations between sulfate and product sulfide (e.g., Canfield et al., 2006). On a local scale, complete H_2_S or S^0^ oxidation could have produced sulfate with δ^34^S values identical to vent fluids, which could have supplied sulfur for MSR. Seawater sulfate varied throughout the Phanerozoic, with estimates of 5–10 mmol/kg H_2_O sulfate at the end Ordovician (Berner, 2004; Horita et al., 2002). The δ^34^S of seawater sulfate at the end Ordovician was around 25–27‰ (Fike et al., 2015, and references therein). Therefore, if seawater sulfate provided an additional substrate for MSR, our values for ancient vent fossils (Figure 5c,d) could actually represent fairly large fractionations of 25‰ to 36‰ by MSR. However, as above, precipitation of sulfides even without microbial influence strongly suggests that hydrogen sulfide was the dominant source of sulfur to the system. This is also supported by our finding of similar δ^34^S values for both modern and Ordovician‐early Silurian vent samples.

Sulfur‐oxidizing bacteria are particularly common at vents (Dick, 2019), and often colonize the tube surfaces of vent tubeworms such as *Alvinella* sp. and *Ridgeia piscesae* (Campbell et al., 2003; Kalanetra & Nelson, 2010). Sulfide oxidizers can produce small isotopic depletions in the resulting product (Balci et al., 2007; Lewis & Roy Krouse, 1968; Nakai & Jensen, 1964). Sulfide oxidizers can also produce sulfur products that are enriched in ^34^S relative to the reactant (Brunner et al., 2008; Pisapia et al., 2007; Zerkle et al., 2009, 2016). Given the latter, sulfide‐oxidizing bacteria colonizing the tubes of modern and ancient vent tubeworms could be producing S^0^ that is heavier in δ^34^S, which would leave pyrite forming from vent fluid with a more depleted δ^34^S signature and thus may produce the sulfur isotope fractionations observed in our samples (Figure 5). S^0^ is a by‐product of microbial sulfide oxidation at vents (Stein et al., 1988; Vetter, 1985) which has also been observed in association with the tubes of *Alvinella* sp., but does not appear to fossilize well (Georgieva et al., 2015). This suggests that the S^0^ left by microbial sulfide oxidation was lost to the system, perhaps being further oxidized to sulfate which diffused out and was diluted by the marine sulfate pool. Alternatively, S^0^ could have undergone disproportionation to produce both sulfate and sulfide, if the sulfur cycling community was complex (e.g., Pellerin et al., 2019). However, disproportionation is generally associated with much larger sulfur isotope fractionation effects (in the region of 20‰–30‰) than those observed in this study. As hydrothermal vents and particularly the surfaces of animal structures in these settings often host complex microbial communities capable of diverse metabolisms (Campbell et al., 2003; Lopez‐Garcia et al., 2002), the δ^34^S values of microbially textured pyrite in this study likely represent a combination of all of the above complex microbial sulfur cycling processes.

It is also possible that negative δ^34^S values of porous pyrite, such as those observed in two of our samples (Figure 5b,d) also represent a contribution from microbial metabolic processes; however, given the absence of fossils in this pyrite, it is difficult to be sure. As microbial fossils are not always preserved alongside remnants of vent animals, further analysis of porous pyrite to establish its mode of formation would be highly beneficial as it may help to recognize microbial processing even in the absence of microbial fossils.

## 6 CONCLUSION

In this study, we were able to observe consistent δ^34^S signatures in pyrites from the recently‐mineralized tubes of two annelid species from two distinct modern vent sites, as well as from two tubeworm fossils from the Ordovician‐early Silurian Yaman Kasy VMS deposit. In all cases, ancient or modern, we observed ^34^S depletion of pyrites containing microbial fossils in comparison with adjacent pyrites not containing microbial fossils averaging approximately 3‰. Our results represent the first indication that the presence of microbial sulfur cycling communities active at the time of pyrite formation influence the sulfur isotope signature of pyrite, imparting a distinct ^34^S depletion, and suggesting that associated pyrite may have also preserved traces of their metabolisms. We interpret these signatures to have resulted from a combination of sulfur cycling processes, including sulfide or elemental sulfur oxidation, hydrogenotrophic sulfate reduction, and/or heterotrophic sulfate reduction by hyperthermophiles. While these findings require further investigation to elucidate the microbial metabolic pathways responsible for the observed sulfur isotope signatures, our findings unearth new possibilities to explore the functioning of recent (e.g., at inactive vent fields) and ancient vent communities, such as the nature of associations between ancient vent animals and microbes.

## Supporting information


File S1

File S2

File S3

File S4

## Data Availability

All data discussed in the paper are present in the main text and [Link], [Link].
