# Supplementary material for: Sulfur isotopes of hydrothermal vent fossils and insights into microbial sulfur cycling within a lower Paleozoic (Ordovician‐early Silurian) vent community
Source: Geobiology. 2022 May 18;20(4):465–78. doi: 10.1111/gbi.12495 (PMC9320992; doi:10.1111/gbi.12495)
Supplement: Supplementary file 1 — File S1 [file GBI-20-465-s002.docx]

**Supplementary File 1: Additional Figures**


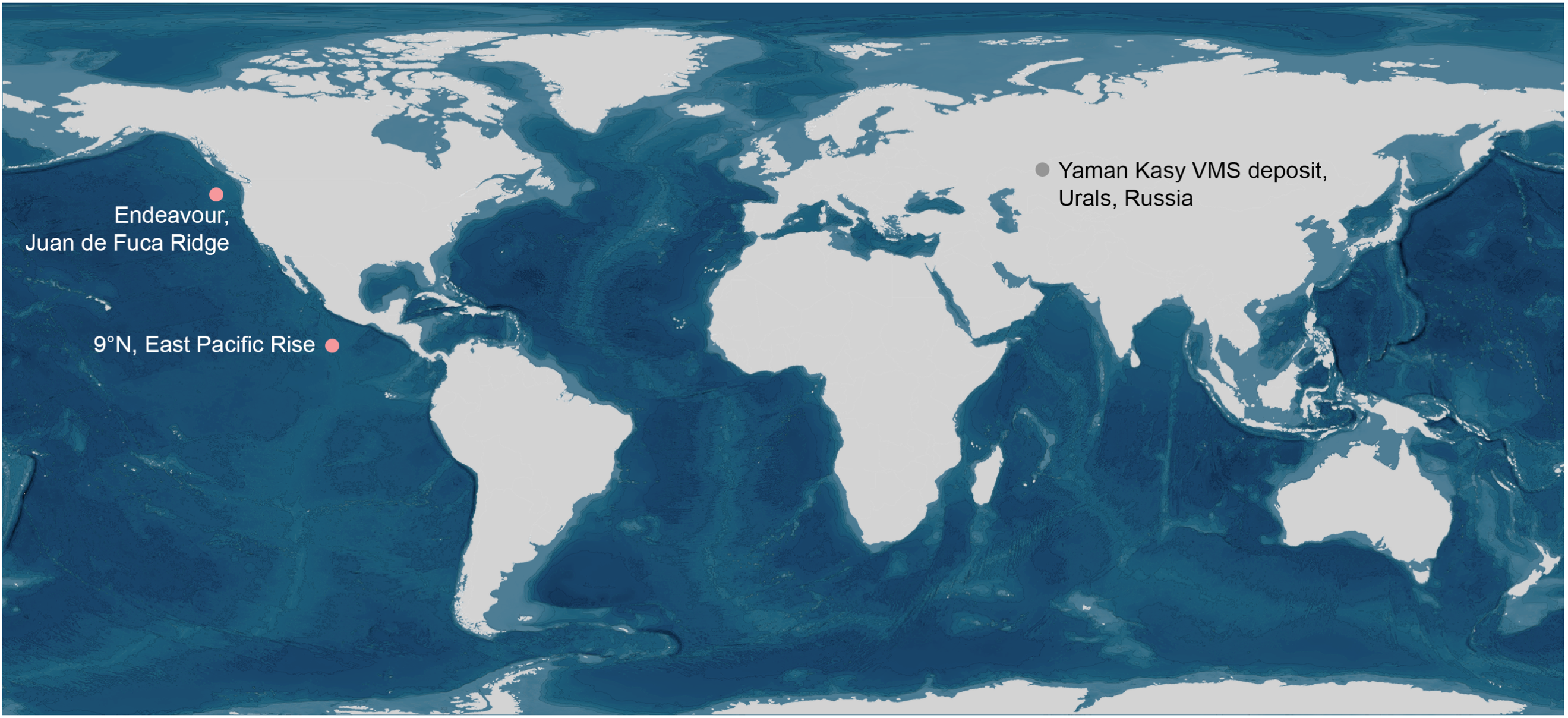


**Figure S1:** Locations of modern day vent sites (pink points) and the ancient fossil VMS deposit (grey point) from which samples were collected. Bathymetry data source: Amante & Eakins (2009).


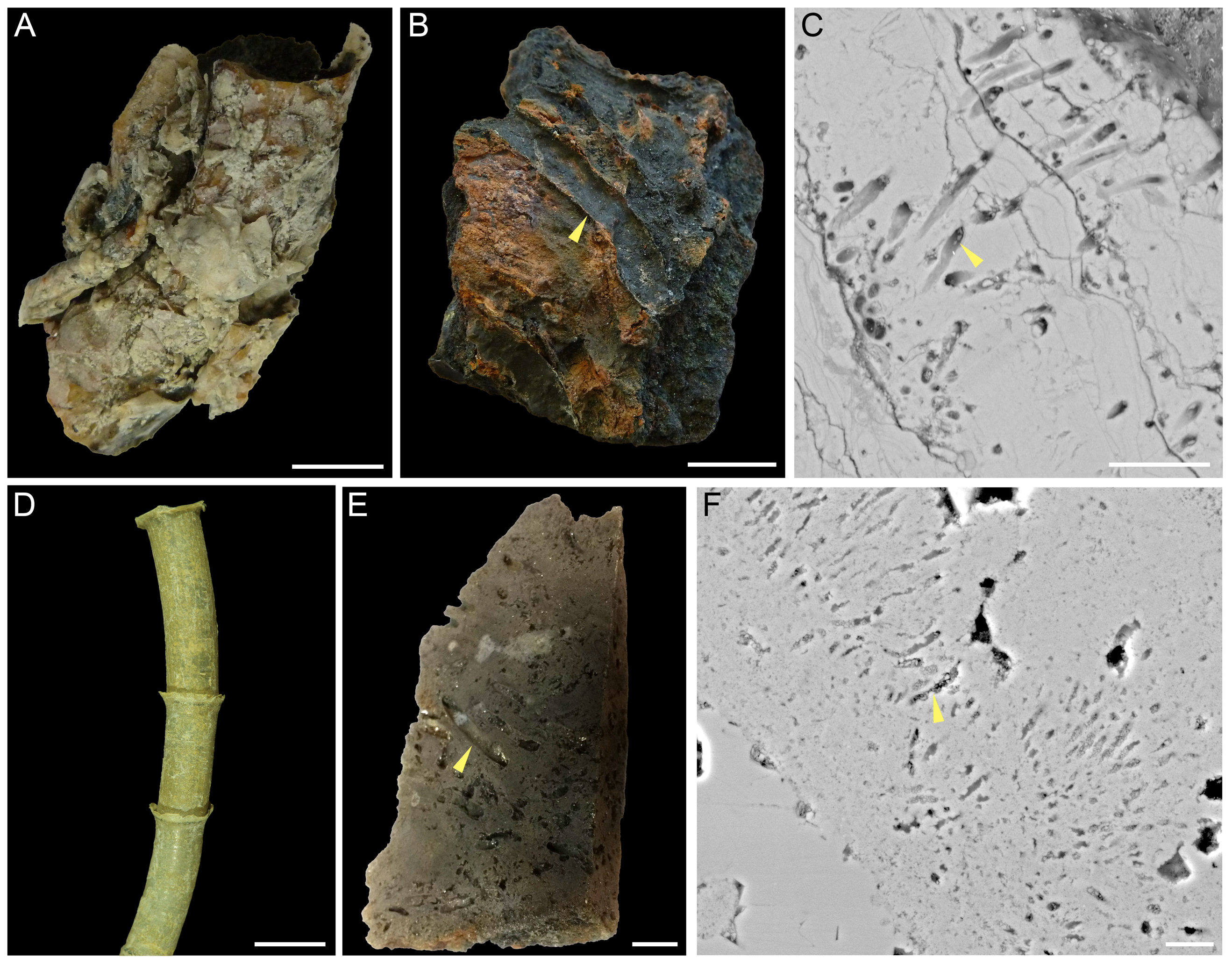


**Figure S2:** Hand specimens of recently-mineralized annelid tubes, and high-resolution images of the microbial fossils preserved within them. A-C, *Alvinella* sp. tubes from 9°N East Pacific Rise; D-F, *Ridgeia piscesae* tubes from Endeavour, Juan de Fuca Ridge. **A**, an unmineralized *Alvinella* sp. tube (not actual specimen used in this study), scale bar is 10 mm. **B**, mineralized *Alvinella* sp. tubes from which sample P23671 used in this study was prepared, scale bar is 20 mm. Yellow arrow points to a mineralized tube. **C**, SEM image of microbial fossils preserved within a mineralized *Alvinella* sp. tube wall in sample P23671, scale bar is 5 µm. Yellow arrow points to a fossil microbial filament. **D**, anterior portion of an unmineralized *R. piscesae* tube (not actual specimen used in this study), scale bar is 5 mm. **E**, mineralized *R. piscesae* tubes from which sample P23672 used in this study was prepared, scale bar is 10 mm. Yellow arrow points to a portion of a tube in longitudinal section. **F**, SEM image of microbial fossils preserved within a mineralized *R. piscesae* tube wall in sample P23672, scale bar is 4 µm. Yellow arrow points to a fossil microbial filament.


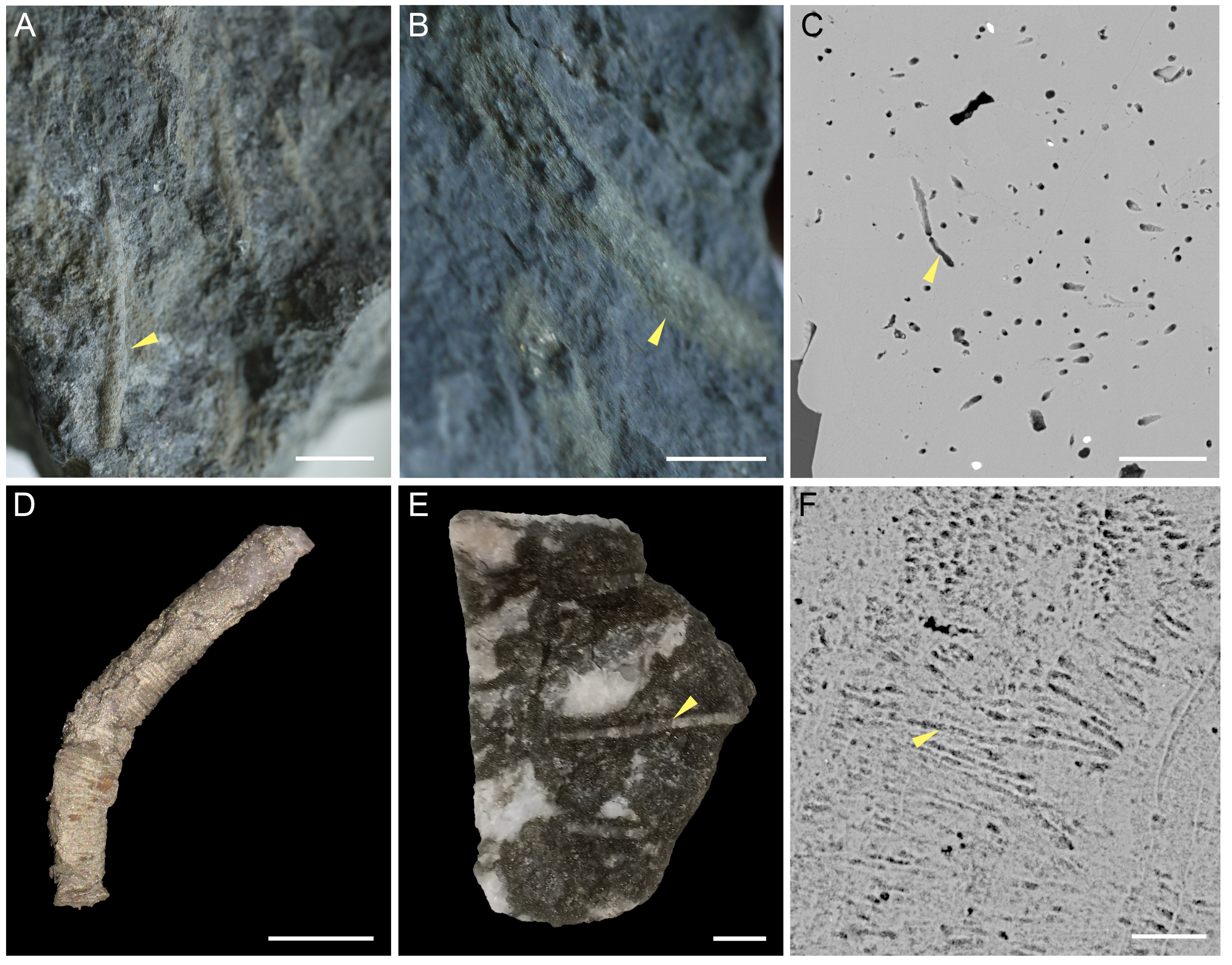


**Figure S3:** Hand specimens of ancient fossil annelid tubes from the Yaman Kasy VMS deposit, Ural Mountains, Russia, and high-resolution images of the microbial fossils preserved within them. A-C, *Yamankasia rifeia* tubes; D-F, *Eoalvinellodes annulatus* tubes. **A**, fossil *Y. rifeia* tubes (not actual specimen used in this study), scale bar is 10 mm. Yellow arrow points to a mineralized tube. **B**, mineralized *Y. rifeia* tube (not actual specimen used in this study) in greater detail (yellow arrow), scale bar is 4 mm. **C**, SEM image of microbial fossils preserved within a *Y. rifeia* fossil tube wall in sample P23934, scale bar is 10 µm. Yellow arrow points to a fossil microbial filament. **D**, fossil *E. annulatus* tube (not actual specimen used in this study), scale bar is 3 mm. **E**, fossil *E. annulatus* tube in longitudinal section (yellow arrow) from which sample P23935 used in this study was prepared, scale bar is 5 mm. **F**, SEM image of microbial fossils preserved within a *E. annulatus* fossil tube wall in sample P23935, scale bar is 10 µm.

**References**

Amante C, Eakins BW (2009) ETOPO1 1-arc-minute global relief model: procedures, data, sources and analysis. *NOAA Technical Memorandum NESDIS NGDC-24* 1–19.
