# Supplementary material for: Sulfur isotopes of hydrothermal vent fossils and insights into microbial sulfur cycling within a lower Paleozoic (Ordovician‐early Silurian) vent community
Source: Geobiology. 2022 May 18;20(4):465–78. doi: 10.1111/gbi.12495 (PMC9320992; doi:10.1111/gbi.12495)
Supplement: Supplementary file 2 — File S2 [file GBI-20-465-s004.doc]

**Supplementary File 2:** Details of the SIMS instrument analytical conditions used for analysis.

**Instrument:** Cameca ims 1270

ACQUISITION PARAMETERS:

| Species | Mass | B Field | Offset | C Time | W Time | Detector |
| --- | --- | --- | --- | --- | --- | --- |
| 32S | 31.972070 | 1326545 | 0.0 | 4.00 | 0.50 | L'2 |
| 33S | 32.971456 | 1326545 | 0.0 | 4.00 | 0.50 | H1 |
| 34S | 33.967866 | 1326545 | 0.0 | 4.00 | 0.50 | H'2 |

ANALYTICAL PARAMETERS:

Sample HV (V) -10,000

Field App. (um) 2511

Entr. Slit (um) 72

Exit Slit (um) 311

Energy Slit (eV) 61

Raster Size (um) 0

Cont. Aperture (um) 400

MRP (mono) 3857 – for Mono Collector, MultiCollector was 4800.

Pressure (mb) 6.6E-9

PRIMARY Ion Species Cs+

Primary HV (V) +10,000

Gun Pressure (mbar) 6.4E-8

L4 Aperture (um) 200

Primary Beam (A) 5.8E-9

ACQUISITION CONTROL PARAMETERS:

Pre-sputtering SELECTED

Reference Signal NOT SELECTED

Mass Calibration Control NOT SELECTED

Energy Control NOT SELECTED

Beam Centering SELECTED

EM HV ADJUST N/A

DETECTOR PARAMETERS

| Detector | Yield | Back Ground | Deadtime |
| --- | --- | --- | --- |
| FC1 | 1.000000 | 1362303 | 0.0 |
| EM | 0.987600 | 0 | 51.0 |
| FC2 | 1.000000 | -79891 | 0.0 |
| L'2 | 0.962357 | -390299 | 0.0 |
| L2 | 1.000000 | 0 | 51.0 |
| L1 | 1.018989 | 72151 | 0.0 |
| C | 0.938000 | 0 | 51.0 |
| H1 | 1.009650 | -69904 | 0.0 |
| H2 | 0.950000 | 0 | 51.0 |
| H'2 | 1.009004 | -91500 | 0.0 |

Pre-Sputter Parameters

| Selected | Duration (Seconds) | Raster Size Start (um) | Raster Size End (um) | Beam Selection Start | Bean Selection End |
| --- | --- | --- | --- | --- | --- |
| True | 60 | 0 | 0 | 5nA | 5nA |

BEAM CENTERING:

| Selected | Field Aperture | Entrance Slits | Contrast Aperture | Option1 | Option2 |
| --- | --- | --- | --- | --- | --- |
| True | yes | no | no |  |  |


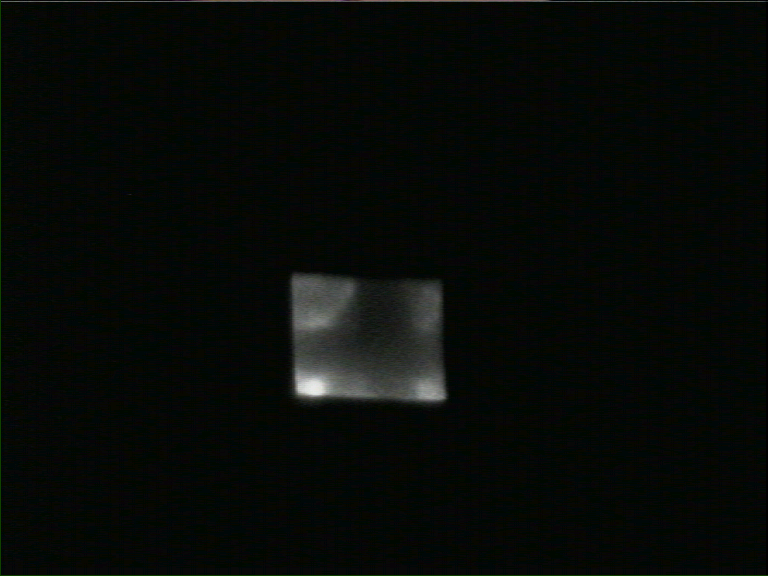


Cameca IMS 1270 aperature.
